# Clinical Outcomes in Early-Stage HER2-Low and HER2-Zero Breast Cancer: Single-Center Experience

**DOI:** 10.3390/jcm14092937

**Published:** 2025-04-24

**Authors:** Jamshid Hamdard, Mehmet Haluk Yücel, Harun Muğlu, Özgür Açıkgöz, Aslı Çakır, Ahmet Bilici, Ömer Fatih Ölmez

**Affiliations:** 1 Faculty of Medicine, Medical Oncology Department, Medipol University, 34214 İstanbul, Türkiye; mhalukyucel@gmail.com (M.H.Y.); hm1635@hotmail.com (H.M.); ozgur_acikgoz@yahoo.com (Ö.A.); ahmetknower@yahoo.com (A.B.); olmezof@gmail.com (Ö.F.Ö.); 2 Faculty of Medicine, Medical Pathology Department, Medipol University, 34214 İstanbul, Türkiye; asli.cakir@medipol.com.tr

**Keywords:** breast cancer, HER2 low, HER2 zero, disease-free survival, overall survival

## Abstract

**Background/Objectives:** The goal of this study is to characterize the survival patterns and outcomes of women with early-stage breast cancer, with a particular emphasis on the distinction between HER2-low and HER2-zero expression. There is limited real-world data on how patients with HER2-negative or HER2-low metastatic or recurrent breast cancer are treated. **Methods:** We retrospectively analyzed the medical records of 1500 breast cancer patients diagnosed between January 2020 and December 2024. From this cohort, 99 patients with HER2-low and 34 patients with HER2-zero early-stage breast cancer were included in our analysis. HER2 low was defined as Immunohistochemistry (IHC) 1+ or IHC 2+ with negative Silver In situ Hybridization (SISH), while HER2 zero was defined as IHC 0. Statistical analyses, including Kaplan–Meier survival analyses and log-rank tests for group comparisons, were performed using IBM SPSS Statistics. **Results:** The median age of patients was 55 years. The HER2-zero group exhibited a higher incidence of brain, liver, bone, and lung metastases (*p* < 0.001 for all) and increased use of CDK4/6 inhibitors (*p* < 0.001). In univariate analyses, younger age, an HER2-zero status, and the absence of metastases were associated with improved disease-free survival (DFS) and overall survival (OS). However, in multivariate analyses, an HER2-zero status independently predicted longer DFS (HR = 0.14, 95% CI: 0.05–0.41, *p* < 0.001) and OS (HR = 0.16, 95% CI: 0.042–0.6, *p* = 0.007). **Conclusions:** Our study revealed distinct metastatic patterns and survival outcomes between HER2-low and HER2-zero early-stage breast cancers. Despite a higher metastatic burden in univariate analyses, HER2 zero status was independently associated with longer DFS and OS in multivariate analyses, highlighting their biological heterogeneity and the need for further research to inform tailored strategies.

## 1. Introduction

Breast cancer (BC) is recognized globally as a significant cause of cancer-related morbidity and mortality [1,2]. The impact of this disease is also considerable in Turkey, where in 2022, approximately 25,249 new cases were diagnosed among women, and 7360 deaths occurred [3]. These figures correspond to 10.5% of all new cancer diagnoses and 5.7% of all cancer-related deaths in the Turkish female population for that year [3].

Breast cancer is not a single disease but a group of distinct subtypes, each with unique molecular and pathological features [4,5,6]. These subtypes respond differently to various treatments [7]. This heterogeneity within breast cancer makes it difficult for clinicians to accurately classify tumors and choose the most effective treatments. Subtypes are categorized by the activity of specific receptors like the estrogen receptor (ER), progesterone receptor (PR), and human epidermal growth factor receptor 2 (HER2). These subtypes differ in risk factors, how they are managed, their outlook, the chance of recurrence, and overall outcomes [8].

The introduction of treatments targeting HER2 has resulted in considerable advancements in the outcomes for patients diagnosed with HER2-positive breast cancer (as determined by IHC 3+ or IHC 2+ with a positive ISH result) [9]. Because of this, HER2 testing is now a standard part of breast cancer diagnosis [10]. However, the field is moving beyond simply classifying tumors as HER2-positive or HER2-negative (IHC 0 or 1+, or IHC 2+ with a negative ISH result). There is now a focus on identifying the “HER2-low” subtype (IHC 1+ or IHC 2+ with a negative ISH result), for which new treatments have been approved in several regions, and others are being actively studied [11,12,13,14,15,16,17,18,19,20].

Recent research indicates that patients with HER2-low tumors may share some clinical characteristics with HER2-positive patients and could benefit from newer HER2-targeted antibody-drug conjugates [21]. The phase III DESTINY-Breast04 trial demonstrated that trastuzumab deruxtecan (T-DXd) significantly extended progression-free survival (PFS) by 4.8 months and OS by 6.6 months compared to standard chemotherapy in patients with HER2-low, advanced breast cancer [22]. Another phase III study, DESTINY-Breast06, compared T-DXd with chemotherapy in HER2-low, hormone receptor-positive metastatic breast cancer that had progressed after hormone therapy [23]. Additionally, a phase II trial is investigating another antibody–drug conjugate, MRG002, in patients with HER2-low, locally advanced, or metastatic breast cancer [24].

Although many studies have examined breast cancer treatment broadly, there is limited real-world data on how patients with HER2-zero (IHC 0) or HER2-low metastatic or recurrent breast cancer are treated. This study aimed to describe the survival patterns and outcomes for women diagnosed with early-stage breast cancer between 2020 and 2024, specifically focusing on differences based on HER2 expression (HER2 low or HER2 zero).

## 2. Materials and Methods

### 2.1. Study Patients

We retrospectively reviewed the medical records of 1500 breast cancer patients diagnosed from January 2020 to December 2024. Of these, 99 HER2-low and 34 HER2-zero early-stage breast cancer patients were enrolled and retrospectively analyzed.

Data on baseline characteristics such as sex, age, site of primary tumor, histopathological type, stage at diagnosis, menopausal status, HER2 status, Ki-67, grade, the presence of brain, liver, bone, and lung metastases, and systemic treatment with CDK4/6 inhibitors were recorded after written informed consent was collected. All patients with metastatic breast cancer older than 18 years of age were included in the study. Patients with histopathology of invasive ductal carcinoma and invasive lobular carcinoma were included in this study. Patients who were HER2 IHC 3-positive, IHC 2-positive, or SISH-positive were excluded.

HER2 zero was defined as IHC 0, and HER2 low was defined as IHC 1+ or IHC2+ with negative SISH.

For systemic neoadjuvant/adjuvant therapy, our patients were treated with cyclophosphamide, anthracycline, and paclitaxel. Hormonal treatment included tamoxifen for premenopausal patients and aromatase inhibitors for postmenopausal patients. The duration of adjuvant hormonal therapy in our patients generally ranged from 5 to 10 years, influenced by individual patient and tumor factors

### 2.2. Statistical Analysis

IBM SPSS Statistics version 27.0 was used for all statistical analyses. The baseline characteristics were summarized using descriptive statistics. The Kaplan–Meier method was used for survival analyses, and the log-rank test was used for group comparisons. DFS was defined as the interval from initial diagnosis to either disease recurrence or death, and OS was defined as the interval from initial diagnosis to death. Univariate analyses assessed the prognostic significance of clinicopathological features. Clinicopathological variables that were significant in univariate analysis or potentially high risk for survival were included in multivariate Cox regression analysis to determine whether they were independent prognostic factors. Hazard ratios (HRs) and their 95% confidence intervals (CIs) were calculated using Cox regression. Continuous data are presented as the mean (standard deviation, SD) or median (range), while categorical data are presented as percentages, as appropriate. Confidence intervals (95% CIs) are also provided where relevant. All reported *p*-values are two-sided, and statistical significance was defined as *p* < 0.05. The data supporting the findings of this study can be obtained from the corresponding author upon request.

## 3. Results

### 3.1. Patient Demographics and Clinical Characteristics

The median age of the patients was 55 years (range 30–90). A total of 18 patients (13.5%) were under 40 years old, 67 patients (50.4%) were between 40 and 60 years old, and 48 patients (36.1%) were 60 years and older. The majority of patients (97.7%, n = 130) were female, and 3 (2.3%) were male. Seventy-three patients (54.9%) were premenopausal, and 60 patients (45.1%) were postmenopausal. In total, 25.6% of patients (n = 34) were HER2 zero, and 74.4% of patients (n = 99) were HER2 low. The Ki-67 level was <20% in 43 patients (32.3%) and ≥20% in 84 patients (63.2%). At the time of diagnosis, 8.3% of the patients (n = 11) were stage 1, 42.1% (n = 56) were stage 2, and 49.6% (n = 66) were stage 3. During follow-up, 9 patients (6.8%) developed brain metastases, 23 patients (17.3%) developed liver metastases, 37 patients (27.8%) developed bone metastases, and 19 patients (14.3%) developed lung metastases. The majority of patients had grade 2 (n = 84, 63.2%) and grade 3 (n = 30, 22.6%) tumors. The most predominant histological type was invasive ductal carcinoma (n = 121, 91%). In total, 95.5% of the patients (n = 127) were ER-positive, and 85% of patients (n = 113) were PR-positive.

There were statistically significant differences in the incidence of brain, liver, bone, and lung metastases between the HER2-zero and HER2-low groups (*p* < 0.001 for all). The incidence of brain, liver, bone, and lung metastases was significantly higher in the HER2-zero group than in the HER2-low group. Additionally, there was a statistically significant difference in the frequency of CDK4/6 inhibitor therapy between the HER2-zero and HER2-low groups (*p* < 0.001). CDK4/6 inhibitor therapy was significantly more frequent in the HER2-zero group than in the HER2-low group. Baseline characteristics are shown in Table 1.

### 3.2. Survival Analysis

**Figure 1:**
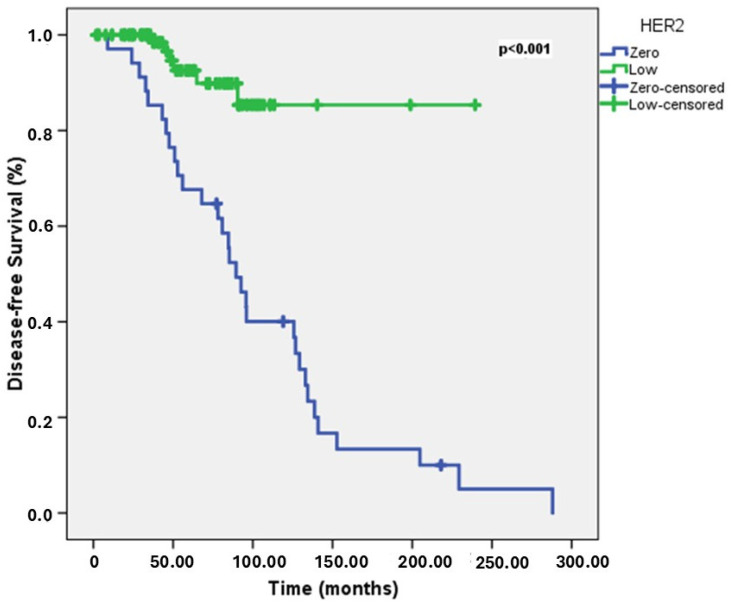
DFS curves in HER2-low vs. HER2-Zero groups.

The median DFS was 133 months (range: 124.3–141.6 months). In the univariate analyses for DFS, age, HER2 status (zero vs. low), the presence of brain, liver, bone, and lung metastasis, and CDK 4/6 inhibitor treatment were found to be significant prognostic factors. Younger patients had a shorter 5-year DFS rate (45.5% vs. 87.4% vs 86.6%, *p* = 0.009). The 5-year DFS rate was 67.6% for the HER2-zero group and 92.6% for the HER2-low group. HER2-zero patients had a longer DFS (*p* = 0.025) (Figure 1). Patients with brain metastases had a 5-year DFS rate of 55.6%, compared to 86.4% for those without brain metastases. The presence of brain metastases was associated with shorter DFS (*p* = 0.003). Patients with liver metastases had a 5-year DFS rate of 60.9%, compared to 91.1% for those without liver metastases. The presence of liver metastases was associated with shorter DFS (*p* < 0.001). Patients with bone metastases had a 5-year DFS rate of 67.5% compared to 93.8% for those without bone metastases. The presence of bone metastases was associated with shorter DFS (*p* < 0.001). Patients with lung metastases had a 5-year DFS rate of 68.4% compared to 87.2% for those without lung metastases. The presence of lung metastases was associated with shorter DFS (*p* = 0.006). Patients receiving CDK4/6 inhibitor therapy had a 5-year DFS rate of 66.6% compared to 95% for those not receiving it. CDK4/6 inhibitor therapy was associated with shorter DFS (*p* < 0.001). In the multivariate analyses for DFS, age, and HER2 status were identified as independent prognostic indicators. Patients under 40 years old had shorter DFS than other age groups (*p* = 0.01, HR = 0.38, 95% CI: 0.19–0.79). HER2-zero patients had longer DFS than HER2-low patients (*p* < 0.001, HR = 0.14, 95% CI: 0.05–0.41). Univariate and multivariate analyses for DFS are summarized in Table 2.

**Figure 2:**
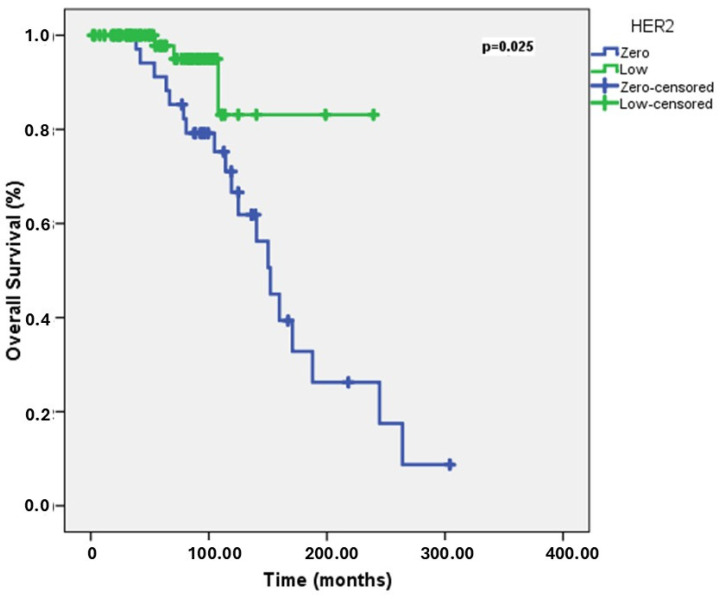
OS curves in HER2-low vs. HER2-Zero groups.

The median OS was 159.7 months (range: 134.4–185.12 months). Within the scope of our study, the median follow-up time was 172.5 months. In the univariate analyses for OS, the 5-year OS rate was 66.7% for patients <40 years old, 95.5% for patients aged 40–60 years, and 94.3% for patients ≥ 60 years. Younger patients had shorter OS (*p* = 0.04). The 5-year OS rate was 91.2% for the HER2-zero group, while it was 97.8% for the HER2-low group. HER2-zero patients had longer OS (*p* = 0.025) (Figure 2). The 5-year OS rate was 88.9% for patients with brain metastases and 96.2% for those without brain metastases. The presence of brain metastases was associated with shorter OS (*p* = 0.025). The 5-year OS rate was 87% for patients with liver metastases and 98.2% for those without liver metastases. The presence of liver metastases was associated with shorter OS (*p* < 0.001). The 5-year OS rate was 89.2% for patients with bone metastases, while this survival rate was not reached in those without bone metastases. The presence of bone metastases was associated with shorter OS (*p* < 0.001). The 5-year OS rate was 89.5% for patients with lung metastases and 97% for those without lung metastases. The presence of lung metastases was associated with shorter OS (*p* = 0.017). The 5-year OS rate was 94.4% for patients receiving CDK4/6 inhibitor therapy, while it was 96% for those not receiving inhibitor therapy. CDK4/6 inhibitor therapy was associated with shorter OS (*p* = 0.025). In multivariate analyses for OS, only HER2 status was an independent risk factor (*p* = 0.007). HER2-zero patients had longer OS than HER2-low patients (HR = 0.16, 95% CI: 0.042–0.6). Univariate and multivariate analyses for OS are shown in Table 3.

## 4. Discussion

This research explored the clinicopathological attributes and prognostic outcomes in women diagnosed with early-stage breast cancer with HER2-low or HER2-zero expressions. Our findings revealed significant differences in metastatic patterns and CDK4/6 inhibitor usage between these two groups. Specifically, HER2-zero patients exhibited a higher incidence of brain, liver, bone, and lung metastases and a greater utilization of CDK4/6 inhibitors compared to HER2-low patients.

Our data indicate that while baseline clinical features such as age, menopausal status, and hormone receptor status did not differ significantly between the HER2-low and HER2-zero groups, the patterns of metastasis did. This suggests that HER2-low status may confer distinct biological properties impacting metastatic spread.

In terms of survival outcomes, our univariate analyses demonstrated that younger age (<40 years), HER2-zero status, and the absence of brain, liver, bone, and lung metastases were associated with improved DFS and OS. Furthermore, the use of CDK4/6 inhibitors was associated with worse DFS and OS in univariate analyses. However, in multivariate analyses, only age and HER2 status remained independent prognostic factors for DFS, with the HER2-zero status independently predicting better OS.

These findings are consistent with recent studies highlighting the prognostic implications of HER2-low expression. For instance, Liu et al. (2024) reported differences in site-specific recurrence patterns between HER2-low and HER2-zero early-stage breast cancer, although they found no significant differences in survival outcomes [25]. This underscores the complexity of HER2-low biology and its impact on disease progression.

Our observation of an increased metastatic burden in HER2-zero patients aligns with the notion that HER2-low tumors may exhibit a less aggressive phenotype. Chen et al. (2023) analyzed a large cohort of Chinese breast cancer patients and explored differences between HER2 ultra-low, HER2-null, and HER2-low expression, further contributing to the understanding of this complex classification [26].

While univariate analyses suggested an association between CDK4/6 inhibitor treatment and shorter DFS and OS, this finding was not statistically significant in the multivariate analyses. This discrepancy in the univariate result is likely attributable to confounding by indication, given that CDK4/6 inhibitors are commonly utilized in patients presenting with more aggressive disease or a higher risk of recurrence. The multivariate analyses, which account for various prognostic factors, offer a more precise evaluation of the independent impact of CDK4/6 inhibitor treatment within this patient cohort. Guven and Sahin (2024) conducted a meta-analysis on the association between HER2-low status and survival in patients treated with CDK4/6 inhibitors, highlighting the continued importance of this treatment modality in metastatic breast cancer [27].

However, the prognosis of HER2-low breast cancer remains controversial. Some studies have shown that HER2-low tumors have a better prognosis than HER2-zero tumors [28,29]. The DESTINY-Breast04 trial demonstrated the efficacy of trastuzumab deruxtecan in HER2-low metastatic breast cancer, significantly improving PFS and OS [22]. This has led to a paradigmatic shift in the management of HER2-low breast cancer. Furthermore, the DESTINY-Breast06 trial provided further evidence of the efficacy of trastuzumab deruxtecan after endocrine therapy [23].

Our study’s finding of distinct metastatic patterns between HER2-low and HER2-zero early-stage breast cancer aligns with Liu et al. (2024), who also observed differences in site-specific recurrence [25]. This consistency reinforces the notion that HER2 status influences the biological behavior of early-stage breast cancer, particularly in the dissemination of metastatic disease. However, while Liu et al. (2024) [25] reported no significant differences in survival outcomes (DFS and OS) between HER2-low and HER2-zero patients, our research indicates that HER2-zero status independently predicts worse DFS and OS. This discrepancy could be attributed to variations in patient populations, treatment protocols, and follow-up duration. Notably, our study included patients diagnosed between 2020 and 2024, a period marked by the emergence and increasing use of novel HER2-targeted antibody–drug conjugates (ADCs), such as trastuzumab deruxtecan, which have significantly impacted treatment strategies and potentially contributed to the observed survival differences. These ADCs have demonstrated efficacy in HER2-low metastatic breast cancer and are increasingly being investigated in earlier disease stages.

In the context of triple-negative breast cancer (TNBC), our results offer a contrasting perspective to Liu et al. (2025), who concluded that having an HER2-low status is associated with improved recurrence-free survival (RFS) in TNBC patients [30]. Our study encompassed a broader spectrum of early-stage breast cancer, not focusing specifically on the TNBC subtype. However, our findings indicate that within our cohort, the HER2-zero status, compared to the HER2-low status, is associated with worse outcomes. This difference highlights the heterogeneity of HER2-low breast cancer and suggests that the prognostic role of HER2-low expression may vary significantly depending on the specific molecular subtype. The more favorable outcomes associated with the HER2-low status in Liu et al.’s (2025) TNBC cohort, compared to the less favorable outcomes in our broader cohort, underscore the need for further research to elucidate the complex interplay between HER2-low expression and other tumor characteristics in predicting prognosis and treatment response [30].

Our study, based on existing clinical and pathological data, offers valuable insights; however, future research would benefit from the inclusion of a wider range of inflammatory and tumor markers. This could potentially improve diagnostic accuracy and better clarify the distinct biological characteristics and prognostic implications associated with the HER2-low and HER2-zero subgroups.

While our study includes a considerable follow-up period (median 172.5 months), longer-term data are needed to confirm the durability of the observed survival differences and assess late events in HER2-low and HER2-zero breast cancer. Future studies with extended follow-up will be crucial to fully understand the long-term prognosis of these distinct subgroups.

Our study has several limitations that should be considered when interpreting the results. Firstly, its retrospective nature and single-center design may limit the generalizability of our findings to broader populations. Secondly, the relatively small sample size (n = 133) and the notable imbalance between the HER2-zero (n = 34) and HER2-low (n = 99) groups are limitations that may affect the statistical power of the multivariate analyses and require cautious interpretation. Furthermore, due to financial constraints, comprehensive molecular profiling beyond the standard IHC and SISH for HER2 classification was not performed, which limits the depth of our biological insights. We also acknowledge that detailed data on the type, duration, and compliance of all systemic chemotherapy and endocrine therapy regimens were not available for all patients due to the retrospective data collection. Finally, while our study includes a considerable follow-up period, longer-term data would provide more robust insights into very long-term survival outcomes and late recurrences in these subgroups. Despite these limitations, we believe our study provides valuable real-world data that contribute to the understanding of HER2-low and HER2-zero early-stage breast cancer.

## 5. Conclusions

In conclusion, our study reveals that early-stage, HER2-low, and HER2-zero breast cancers, while falling under the traditional HER2-negative umbrella, demonstrate distinct clinical behaviors. Specifically, we observed differing patterns of metastasis, and, importantly, our multivariate analysis indicated that HER2-zero status was independently associated with longer DFS and OS compared to HER2-low status. These findings underscore the biological heterogeneity within HER2-negative breast cancer and suggest that HER2-zero tumors may have a unique prognosis distinct from HER2-low tumors despite showing a higher metastatic burden in univariate analyses. Further large-scale, prospective studies are essential to validate these observations, delve deeper into the underlying biological mechanisms driving these differences, and ultimately inform the development of more precisely tailored treatment strategies for patients within these specific subgroups.

## Figures and Tables

**Table 1:** Baseline characteristics by chi-square test.

Characteristics	HER2 Zero n (%)	HER2 Low n (%)	*p*-Value
Age			0.247
<40	2 (5.9%)	16 (16.2%)	
40–60	17 (50%)	50 (50%)	
≥60	15 (44.1%)	33 (33.3%)	
Gender			0.755
Female	33 (97.1%)	97 (98%)	
Male	1 (2.9%)	2 (2%)	
Tumor Location			0.408
Right	14 (41.2%)	50 (50.5%)	
Left	20 (58.8%)	47 (47.5%)	
Bilateral	0 (0%)	2 (2%)	
Menopause Status			0.182
Premenopausal	22 (64.7%)	51 (51.5%)	
Postmenopausal	12 (35.3%)	48 (48.5%)	
ER Status			0.609
Positive	33 (97.1%)	94 (94.9%)	
Negative	1 (2.9%)	5 (5.1%)	
PR Status			0.950
Positive	29 (85.3%)	84 (84.8%)	
Negative	5 (14.7%)	15 (15.2%)	
Ki-67 Status			0.492
<20	11 (39.3%)	32 (32.3%)	
≥20	17 (60.7%)	67 (67.7%)	
Histological Grade			0.134
1	0 (0%)	7 (7.1%)	
2	19 (86.4%)	65 (77.4%)	
3	3 (13.6%)	27 (27.3%)	
Stage at Diagnosis			0.078
Stage 1	0 (0%)	11 (11.1%)	
Stage 2	18 (52.9%)	38 (38.4)	
Stage 3	16 (47.1%)	50 (50.5)	
Brain Metastasis			<0.001
Present	9 (26.5%)	0 (0%)	
Absent	25 (73.5%)	99 (100%)	
Liver Metastasis			<0.001
Present	20 (58.8%)	3 (3%)	
Absent	14 (41.2%)	96 (97%)	
Bone Metastasis			<0.001
Present	32 (94.1%)	5 (5.1%)	
Absent	2 (5.9%)	94 (94.9%)	
Lung Metastasis			<0.001
Present	18 (52.9%)	1 (1%)	
Absent	16 (47.1%)	98 (99%)	
CDK4/6 Inhibitor Treatment			<0.001
Present	31 (91.2%)	5 (5.1%)	
Absent	3 (8.8%)	94 (94.9%)	

**Table 2:** Univariate and multivariate analyses results for DFS.

Variables	5-Year DFS Rate (%)	Univariate *p* Value	HR (%95 CI)	Multivariate *p* Value
Age, years		0.009	0.38 (0.19–0.79)	0.010
≤40	45.5			
40–60	87.4			
≥60	86.6			
Gender		0.200		
Female	84			
Male	66.7			
Menopausal Status		0.600		
Premenopausal	83.2			
Postmenopausal	84.5			
ER Status		0.880		
Positive	83			
Negative	50			
PR Status		0.250		
Positive	86.2			
Negative	67.3			
HER2 Status		<0.001	0.14 (0.05–0.41)	<0.001
Zero	67.6			
Low	92.6			
Ki-67 Levels		0.610		
<20	88.3			
≥20	79.9			
Brain Metastases		0.003		
Present	55.6			
Absent	86.4			
Liver Metastases		<0.001		
Present	60.9			
Absent	91.1			
Bone Metastases		<0.001		
Present	67.5			
Absent	93.8			
Lung Metastases		0.006		
Present	68.4			
Absent	87.2			
Tumor Size		0.072	1.26 (0.69–2.28)	0.440
<2cm	93.8			
2–5cm	84.4			
≥5cm	70.6			
Amount of Lymph Node Metastasis		0.510		
0	91.5			
1–3	82			
≥4	80.6			
CDK 4/6 Inhibitor Treatment		<0.001	0.45 (0.14–1.46)	0.180
Present	66.6			
Absent	95			

**Table 3:** Univariate and multivariate analysis results for OS.

Variables	5-Year OS (%)	Univariate *p* Value	HR (%95 CI)	Multivariate *p* Value
Age, years		0.040	0.76 (0.33–1.70)	0.500
≤40	66.7			
40–60	95.5			
≥60	94.3			
Gender		0.100		
Female	95.2			
Male	NR			
Menopausal Status		0.520		
Premenopausal	95.9			
Postmenopausal	94.4			
ER Status		0.510		
Positive	95.1			
Negative	NR			
PR Status		0.064		
Positive	95.8			
Negative	91.7			
HER2 Status		0.025	0.16 (0.042–0.6)	0.007
Zero	91.2			
Low	97.8			
Ki-67 Levels		0.510		
<20	96.7			
≥20	93.7			
Brain Metastases		0.025		
Present	88.9			
Absent	96.2			
Liver Metastases		<0.001		
Present	87			
Absent	98.2			
Bone Metastases		<0.001		
Present	89.2			
Absent	NR			
Lung Metastases		0.017		
Present	89.5			
Absent	97			
Tumor Size		0.440		
<2cm	NR			
2–5cm	93.4			
≥5cm	94.1			
Amount of Lymph Node Metastasis		0.190		
0	95.2			
1–3	NA			
≥4	88.4			
Histologic Type		0.042	1.37 (0.46–4.08)	0.560
Invasive Ductal Carcinoma	98.6			
Invasive Lobuler Carcinoma	70.1			
CDK 4/6 Inhibitor Treatment		0.025	0.82 (0.19–3.57)	0.800
Present	94.4			
Absent	96			

NA: Not applicable NR: Not reached.

## Data Availability

The data supporting the findings of this research are not openly accessible, but interested parties may contact the corresponding author for further information.
